# Identification and Validation of Metabolic Hub Genes Using WGCNA and Prognostic Risk Modeling in Acute Myeloid Leukemia

**DOI:** 10.1002/cnr2.70645

**Published:** 2026-08-03

**Authors:** Samaneh Ramezani, Fatemeh Akhoundi, Reza Valadan, Hossein Asgarian‐Omran

**Affiliations:** ^1^ Department of Immunology, School of Medicine Mazandaran University of Medical Sciences Sari Iran; ^2^ Department of Genetics, Faculty of Basic Sciences Shahrekord University Shahrekord Iran; ^3^ Gastrointestinal Cancer Research Center Non‐Communicable Diseases Institute, Mazandaran University of Medical Sciences Sari Iran

**Keywords:** acute myeloid leukemia, CYP4F3, DNMT3A, G6PD, metabolic reprogramming, PFKL, prognostic signature

## Abstract

**Background:**

Acute myeloid leukemia (AML) is an aggressive and molecularly heterogeneous hematologic malignancy associated with poor clinical outcomes, particularly in elderly and high‐risk patients. Increasing evidence suggests that metabolic reprogramming plays a critical role in leukemia progression, immune dysregulation, and therapeutic resistance. However, the prognostic value of metabolism‐associated molecular networks in AML remains insufficiently understood.

**Aim:**

This study aimed to identify metabolism‐related hub genes involved in AML progression and to establish a robust prognostic signature for survival prediction.

**Methods and Results:**

Integrated transcriptomic analyses were performed using multiple Gene Expression Omnibus datasets and the TCGA‐LAML cohort. Weighted gene co‐expression network analysis identified AML‐associated gene modules, followed by protein–protein interaction and functional enrichment analyses. Metabolic‐related hub genes were selected through integration with curated metabolic gene sets. A prognostic model was subsequently developed using univariable and least absolute shrinkage and selection operator (LASSO) Cox regression analyses and validated in independent cohorts. A four‐gene metabolic signature consisting of CYP4F3, PFKL, G6PD, and DNMT3A was identified as an independent predictor of overall survival. Patients in the high‐risk group showed significantly poorer survival outcomes compared with low‐risk patients (*p* < 0.0001). The prognostic model demonstrated stable and reliable predictive performance across training, testing, and validation cohorts. Functional enrichment analyses revealed that the identified genes are closely associated with metabolic pathways, immune‐related signaling, and leukemic microenvironment remodeling. Notably, increased expression of G6PD and PFKL was associated with adverse prognosis, supporting the contribution of altered glycolysis and redox homeostasis to AML pathogenesis.

**Conclusions:**

Our findings establish a metabolic‐based prognostic signature with strong predictive utility in AML. The identified metabolic hub genes provide insight into the interaction between metabolic dysregulation and immune remodeling in leukemia and may serve as promising biomarkers for risk stratification and potential therapeutic targets.

## 1 Introduction

Acute myeloid leukemia (AML) is a heterogeneous hematologic malignancy characterized by the clonal expansion of abnormal myeloid progenitors in the bone marrow (BM), leading to impaired hematopoiesis and progressive disease [1]. Despite advances in induction chemotherapy based on anthracyclines and cytarabine, long‐term outcomes remain suboptimal, particularly in elderly patients and those with high‐risk cytogenetic profiles [2, 3]. The 5‐year overall survival rate declines markedly with age, underscoring the need for improved molecular stratification and novel therapeutic targets [2]. The bone marrow microenvironment plays a central role in AML pathogenesis by providing structural and metabolic support to leukemic cells [4]. Hematopoietic stem cells (HSCs) exhibit remarkable metabolic plasticity, enabling them to adapt to physiological and pathological stress. Quiescent long‐term HSCs primarily rely on glycolysis to preserve stemness and limit oxidative damage, whereas activated HSCs shift toward oxidative phosphorylation (OXPHOS) to support proliferation and differentiation. These metabolic adaptations are regulated by mitochondrial activity, substrate availability, and interactions with the bone marrow niche, including mitochondrial transfer from stromal cells. In addition, stress‐responsive HSCs enhance fatty acid oxidation and amino acid metabolism through pathways controlled by key regulators such as carnitine palmitoyl transferase 1A (CPT1A), peroxisome proliferator‐activated receptor alpha (PPARα), peroxisome proliferator‐activated receptor gamma coactivator 1‐alpha (PGC1A), branched‐chain amino acid transaminase 1 (BCAT1), AMP‐activated protein kinase (AMPK), and mammalian target of rapamycin (mTOR). These metabolic processes are closely linked to epigenetic regulation mediated by enzymes such as DNA methyltransferase 3 alpha (DNMT3A) [5].

Increasing evidence indicates that metabolic reprogramming, encompassing glycolysis, the pentose phosphate pathway, mitochondrial metabolism, and lipid utilization, not only sustains leukemic cell proliferation but also shapes the immune microenvironment and contributes to therapeutic resistance. Notably, metabolic enzymes such as glucose‐6‐phosphate dehydrogenase (G6PD) and phosphofructokinase liver type (PFKL) regulate redox homeostasis and energy production, while epigenetic modifiers like DNMT3A influence both metabolic and transcriptional networks [6, 7, 8, 9]. However, most previous studies have focused on individual genes or differential expression analyses, limiting the understanding of coordinated regulatory networks underlying AML metabolism [8, 10, 11]. Network‐based approaches offer a systems‐level perspective to address this limitation. Weighted gene co‐expression network analysis (WGCNA) enables the identification of biologically relevant gene modules and highly interconnected hub genes, facilitating the exploration of complex interactions between metabolic pathways and immune signaling. Integrating such approaches with survival modeling can uncover robust prognostic signatures with mechanistic relevance.

In this study, multiple Gene Expression Omnibus (GEO) datasets with The Cancer Genome Atlas (TCGA) data from the Recount2 platform were integrated to construct a comprehensive gene co‐expression network in AML. Through the combination of WGCNA, protein–protein interaction (PPI) analysis, and Cox regression modeling, a set of metabolic‐related hub genes was identified and developed a prognostic risk signature. Notably, our findings reveal that these genes are not only involved in metabolic pathways but are also linked to immune regulation and microenvironment remodeling, highlighting a coordinated role of metabolism and immune signaling in AML progression.

## 2 Materials and Methods

### 2.1 Data Collection and Preprocessing

**FIGURE 1:**
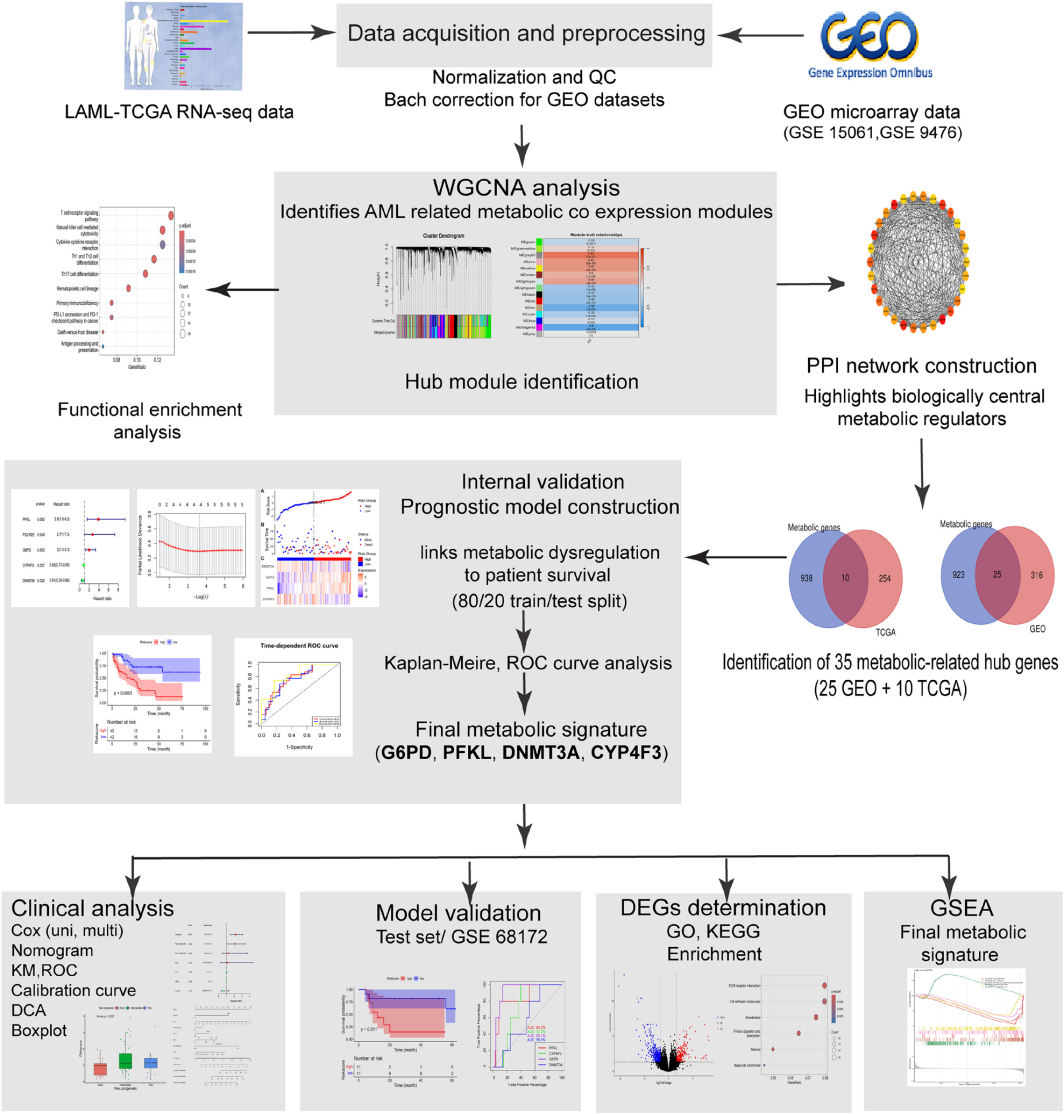
Schematic overview of the study design workflow.

Gene expression and clinical data for AML were obtained from GEO (GSE9476 [6], GSE15061 [7], GSE68172) and TCGA‐LAML via Recount2 platform [8, 9]. GSE9476 and GSE15061 were merged after batch correction using “ComBat” [10], (“sva” package) [11], while GSE68172 served as an external validation cohort. TCGA RNA‐seq data (126 AML, 102 normal) were normalized and filtered using “TCGAbiolinks” [12, 13]. Also, clinical and molecular data for TCGA‐LAML patients were downloaded from the TCGA/cBioPortal public dataset, including the file “laml_tcga_pub_clinical_data.tsv,” which was used to supplement incomplete clinical annotations. Metabolic‐related genes were retrieved from MSigDB (https://www.gseamsigdb.org/gsea/msigdb). The overall workflow of the study is illustrated in Figure 1.

### 2.2 Weighted Gene Co‐Expression Network Analysis

WGCNA [14] was performed on GEO and TCGA datasets. Soft‐thresholding powers were selected based on scale‐free topology criteria (*R* ^2^ > 0.8 for GEO; *R* ^2^ > 0.85 for TCGA). Then, the weighted adjacency matrix was constructed using the soft thresholding power β in order to achieve a scale‐free topology [15]. Gene modules were identified using hierarchical clustering and dynamic tree cutting (minimum size = 30), followed by module merging (cut height = 0.25). Significant modules correlated with AML status (|*r*| > 0.45, *p* < 0.05) were selected.

### 2.3 Functional Enrichment Analysis

Gene ontology (GO) and kyoto encyclopedia (KEGG) enrichment analyses were conducted using “clusterProfiler” [16] with FDR‐adjusted *p* < 0.05 to characterize biological functions of significant modules.

### 2.4 Construction of Protein–Protein Interaction Network

Protein–protein interaction networks were generated using STRING in Cytoscape (Version: 3.10.0) (score ≥ 0.4). Hub genes were identified using CytoHubba [14] based on 11 topological algorithms, and top‐ranked genes were selected.

### 2.5 Identification of the Metabolic‐ Related Hub Genes

Hub genes from GEO and TCGA were separately intersected with MSigDB metabolic gene sets to identify robust metabolic‐related candidates, minimizing platform‐specific bias.

### 2.6 Internal Validation and Evaluation of Metabolic‐Related Hub Genes

TCGA samples were split into training and testing sets (80:20). Prognostic genes were screened using univariable Cox regression and refined by least absolute shrinkage and selection operator (LASSO) Cox analysis. The optimal penalty parameter (λ) was determined by 10‐fold cross‐validation using the cv.glmnet function, and the value corresponding to the minimum cross‐validation error (lambda.min = 0.0269) was selected. The prognostic risk score was calculated by linearly combining the regression coefficients (*β*) obtained from the least absolute shrinkage and selection operator (LASSO) model with the corresponding gene expression levels:

$$\text{Risk score}=\Sigma \;\left(\text{gene expression level}\times \beta \;\text{coefficient}\right).$$

Then, patients were stratified into high‐ and low‐risk groups based on the median risk score. Kaplan–Meier (KM) survival analysis and time‐dependent receiver operating characteristic (ROC) curves were performed to evaluate the predictive performance of the model.

### 2.7 Nomogram Construction and Clinical Data Analysis

To identify prognostic determinants of overall survival (OS), univariable and multivariable Cox analyses were performed for each clinical factor. A nomogram was then constructed for each clinical data using the “rms” package in R to estimate individualized 1‐, 3‐, and 5‐year OS probabilities. Notably, AML patients were classified into three groups: good, intermediate, and poor risk cytogenetic according to chromosomal abnormality patterns reported in the dataset. Threshold probabilities ranging from 0 to 0.5 were examined. To determine the performance and predictive accuracy of the line chart, KM, ROC analysis, calibration curves, and the concordance index (C‐index) were employed. Decision curve analysis (DCA) was used to assess the various predictive models' clinical utility [15]. By measuring the net revenue under various threshold probabilities, it may examine how the nomogram differs from other models. *p* < 0.05 (bilateral) was deemed statistically significant in this investigation.

### 2.8 Identification and Enrichment Analysis of Differentially Expressed Genes (DEGs)

The differentially expressed genes between the high‐ and low‐risk groups in TCGA‐LAML cohort were identified using the “DEseq2” (|log_2_FC| > 1, *p* < 0.05) [17]. To better valuation of the functions of the DEGs in AML, GO and KEGG enrichment analysis were done.

### 2.9 Gene Set Enrichment Analysis (GSEA)

To distinguish biological differences in final hub genes, the GSEA method and the h. all. v2024.1. Hs. symbols gene set were applied. Pathways with nominal *p*‐value < 0.05 and FDR < 0.25 were regarded as statistically significant.

### 2.10 Construction of the Nomogram and Validation of Metabolic‐Related Prognostic Genes (MRPGs)

To facilitate clinical application, a nomogram was constructed based on the final MRPGs to predict 12‐, 36‐, and 60‐month survival probabilities. The prognostic risk model was subsequently evaluated in the internal test group using Kaplan–Meier survival analysis and time‐dependent receiver operating characteristic (ROC) analysis based on the calculated risk score.

In addition, the external GEO dataset GSE68172 was used for independent evaluation of the final hub genes at the gene level. Differential expression analysis and ROC analysis using the “pROC” package were performed for CYP4F3, PFKL, G6PD, and DNMT3A in GSE68172.

### 2.11 Statistical Analysis

All statistical analyses and visualizations were performed using R (version 4.3.2). Statistical significance was defined as *p* < 0.05. WGCNA was constructed using Pearson correlation. Prognostic signature was identified through univariable Cox regression and LASSO analysis. Overall survival was evaluated using Kaplan–Meier curves with the log‐rank test.

## 3 Results

### 3.1 Data Collection and Preprocessing

Batch effect correction effectively integrated the GEO datasets (228 AML and 107 normal samples) by removing technical variation, as evidenced by the intermingling of samples from different batches in the principal component analysis (PCA) space (Figure S1A,B). TCGA‐LAML data included 126 AML and 102 normal samples (Table 1). Also, GSE68172 data was preprocessed, which enrolled in the following analysis as validation dataset (Table 2).

**TABLE 1:** Clinical characteristics of TCGA‐LAML dataset.

Characteristic	*N* = 175
Age at diagnosis	55 ± (16)
Prior malignancy	11 (6.3%)
Gender	
Female	80 (46%)
Male	95 (54%)
Vital status	
Alive	65 (37%)
Dead	110 (63%)
FAB	
M0	14 (8.0%)
M1	41 (23%)
M2	38 (22%)
M3	18 (10%)
M4	39 (22%)
M5	18 (10%)
M6	3 (1.7%)
M7	3 (1.7%)
Unknown	1 (0.6%)
Risk cytogenetic	
Good	35 (20%)
Intermediate	102 (58%)
N.D.	4 (2.3%)
Poor	34 (19%)
WBC	14 (3, 47)
OS	
> 12 months	95 (54%)
< 12 months	80 (46%)

*Note:* Mean ± (SD); *n* (%); Median (Q1, Q3).

Abbreviations: FAB, French–American–British classification; *N*, number; ND, not determined; OS, overall survival; WBC, white blood cell count.

**TABLE 2:** Main information of the GEO datasets used for meta‐analysis and validation.

GEO datasets ID	Experiment type platform	GPL	Number of tumor sample	Number of normal sample	Total sample
GSE9476	Affymetrix Human Genome U133A Array (HG‐U133A)	GPL 96	26	38	64
GSE15061	Affymetrix Human Genome U133 Plus 2.0 Array (HG‐U133_Plus_2)	GPL 570	202	69	271
GSE68172	[HG‐U133_Plus_2] Affymetrix Human Genome U133 Plus 2.0 Array	GPL 570	77	5	72

Abbreviations: GEO, Gene Expression Omnibus; GPL, GEO platform.

### 3.2 Identification of Co‐Expression Modules and Candidate Genes

**FIGURE 2:**
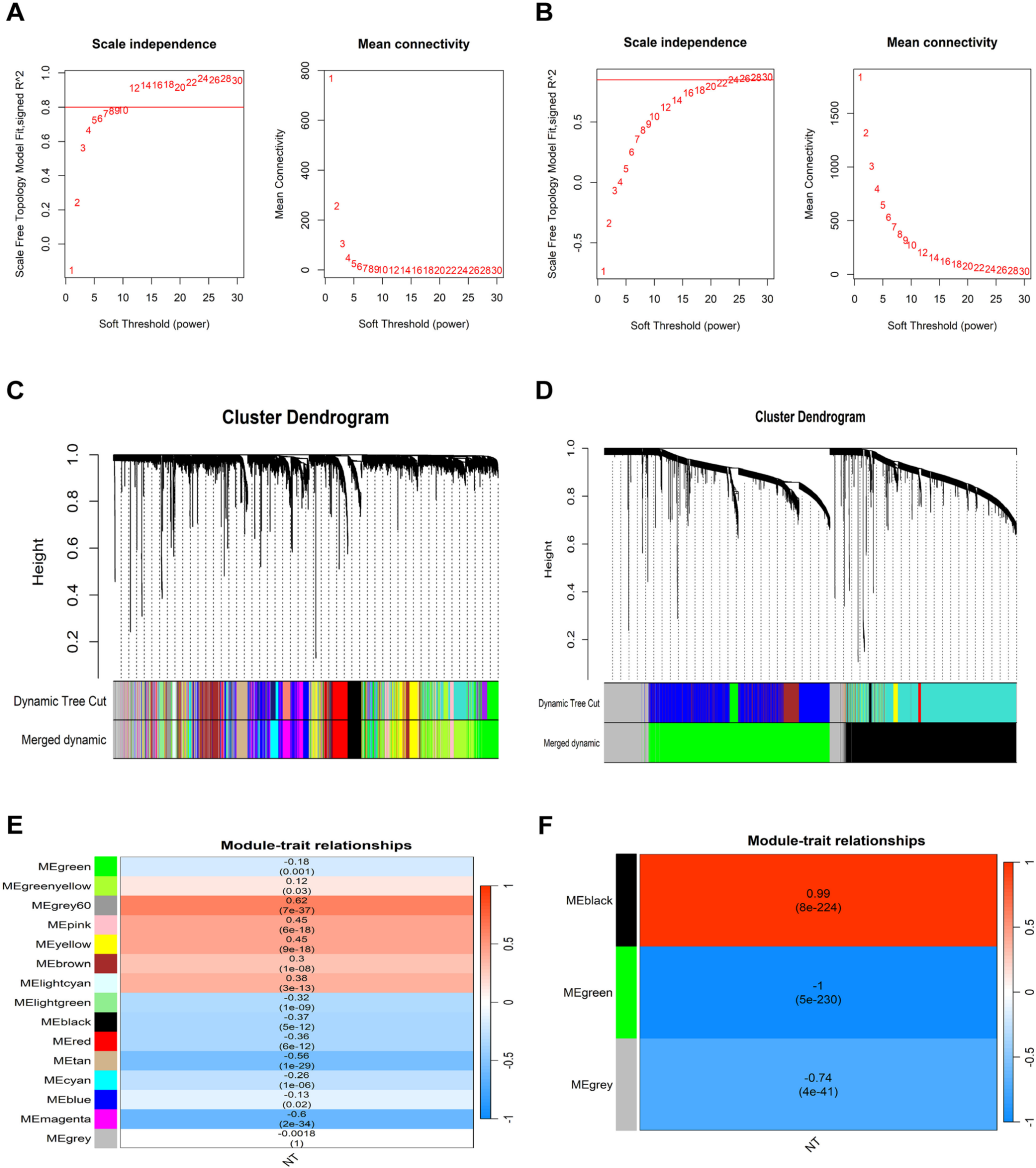
WGCNA identifies biologically relevant gene modules in AML using GEO and TCGA datasets. (A, D) Scale‐free topology fit index and mean connectivity across soft‐thresholding powers. The soft‐thresholding power (*β* = 10 for GEO, 24 for TCGA) was selected as the lowest value achieving approximate scale‐free topology (*R* ^2^ ≥ 0.8 for GEO, *R* ^2^ ≥ 0.85 for TCGA) while preserving network connectivity. (B, E) Hierarchical clustering dendrogram based on topological overlap, with dynamic tree cutting used to define co‐expression modules (color‐coded). (C, F) Module–trait relationships showing correlations between module eigengenes and clinical/metabolic traits in AML. Rows represent eigengenes and columns represent traits. Each cell shows the correlation and *p*‐value. WGCNA clusters co‐expressed genes into modules associated with shared biological functions and dysregulated pathways in AML. AML, acute myeloid leukemia; GEO, Gene Expression Omnibus; TCGA, The Cancer Genome Atlas; WGCNA, weighted gene co‐expression network analysis.

After preprocessing, expression matrices containing 13 237 genes from 335 GEO samples and 18 441 genes from 228 TCGA samples were used as input for WGCNA. The top 6000 genes with the highest variability based on mad scale were selected for network construction. Sample clustering using Euclidean distance revealed no outlier samples in either dataset (Figure S1C,D). Scale‐free topology analysis indicated that the network reached a fitting index above 0.8 at *β* = 10 for the GEO dataset (Figure 2A) and above 0.85 at *β* = 24 for the TCGA dataset (Figure 2B); thus, these soft‐thresholding powers were chosen for subsequent analyses. Mean connectivity remained stable at the selected *β* values. Using these parameters, hierarchical clustering and module eigengene heatmaps were generated (Figure S2A–D). Following module merging based on eigengene dissimilarity, 18 modules were identified in the GEO dataset and 7 modules in the TCGA dataset (Figure S2E,F; Tables S1 and S2). Heatmaps of gene expression patterns for each module are shown in Figure 2C,D. In the GEO module–trait heatmap (Figure 2E,F), two modules demonstrated significant positive correlations with the clinical trait (pink: cor = 0.45, *p* = 6 × 10^−18^; yellow: cor = 0.45, *p* = 9 × 10^−18^), whereas the magenta (cor = −0.60, *p* = 2 × 10^−34^) and tan (cor = −0.56, *p* = 1 × 10^−29^) modules showed the strongest negative associations. In the TCGA dataset, the black and green modules exhibited the highest correlations with AML versus normal samples (black: cor = 0.99, *p* = 8 × 10^−224^; green: cor = −1.00, *p* = 5 × 10^−230^) (Table S3). Therefore, these modules were selected for hub gene identification.

**FIGURE 3:**
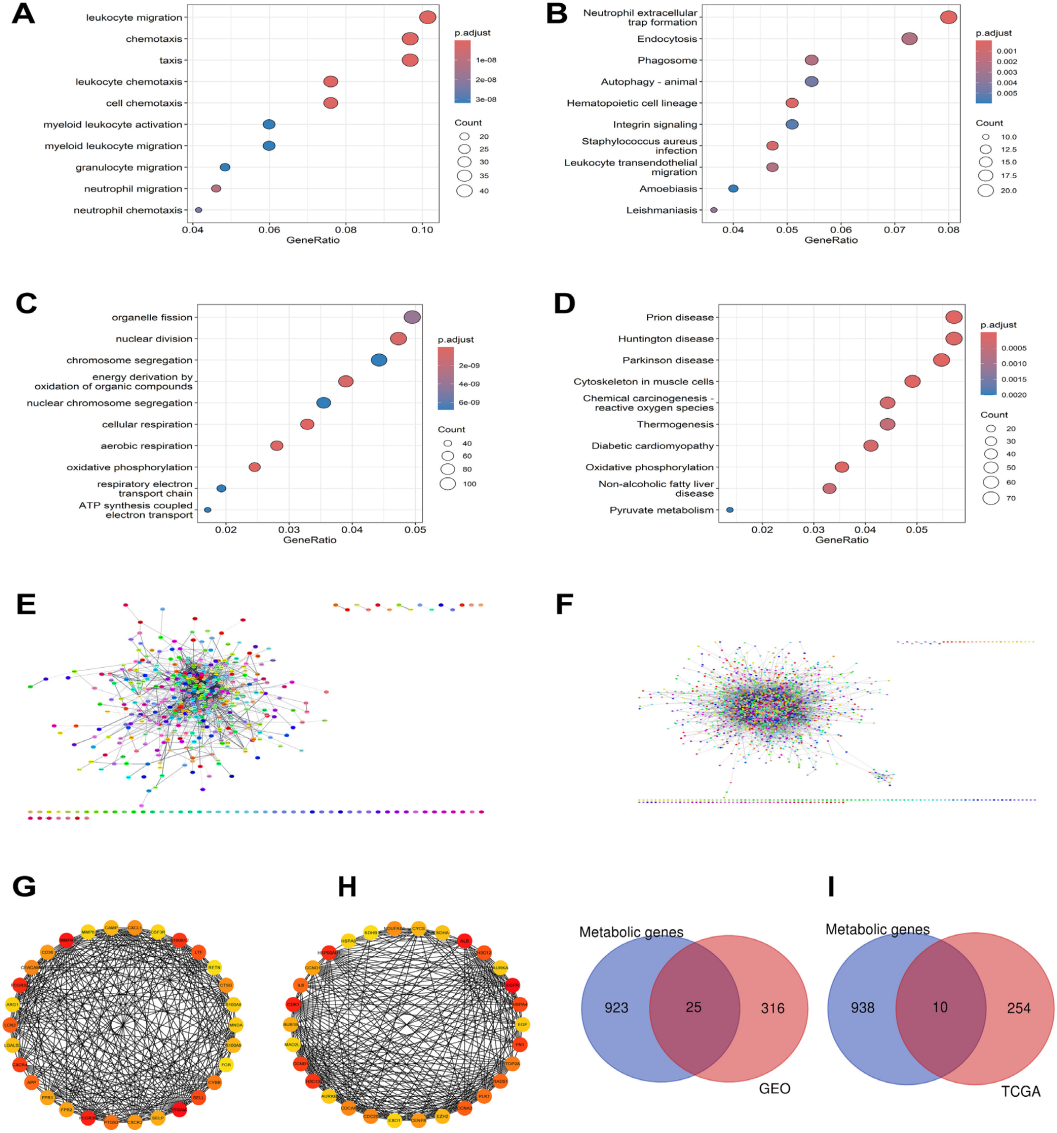
Functional characterization of hub genes derived from GEO and TCGA datasets in AML. (A, B) Gene Ontology of biological processes and KEGG pathway enrichment analyses of 477 genes from GEO data. (C, D) Gene Ontology (biological processes) and KEGG pathway enrichment analyses of 2631 genes from TCGA data. (E, F) PPI networks of module genes identified from GEO (E) and TCGA (F) datasets. (G, H) Top 30 hub genes ranked by degree centrality using 11 CytoHubba algorithms (Cytoscape) for GEO (G, magenta module) and TCGA (H, black module). (I) Venn diagram showing the overlap between hub genes from GEO and TCGA datasets and metabolic‐related genes from MSigDB. These analyses identify key pathways and metabolic processes associated with AML. AML, acute myeloid leukemia; GEO, Gene Expression Omnibus; KEGG, Kyoto Encyclopedia of Genes and Genomes; PPI, Protein–protein interaction; TCGA, The Cancer Genome Atlas.

Subsequently, functional enrichment analysis was performed for the magenta, pink, tan, yellow, black, and green modules using the “ClusterProfiler” package, and the top 10 significantly enriched pathways (*p* < 0.05) were identified. As shown in Figure 3A,B, gene ontology biological process (BP) analysis of the magenta module revealed significant enrichment in leukocyte migration, positive regulation of cytokine production, and leukocyte chemotaxis. KEGG pathway analysis further indicated enrichment in neutrophil extracellular trap formation, endocytosis, and phagosome pathways. For the green module, BP enrichment analysis showed that genes were mainly involved in nuclear division, energy derivation by oxidation of organic compounds, and nuclear chromosome segregation (Figure 3C,D). These findings suggest that dysregulated immune signaling and altered energy metabolism may jointly contribute to AML progression and therapeutic resistance. Detailed enrichment results for the remaining modules are provided in Figures S3 and S4.

To further investigate interactions among module genes, PPI networks were constructed for the magenta, pink, tan, yellow, black, and green modules using the STRING database and visualized in Cytoscape. Eleven topological algorithms were applied using the CytoHubba plugin, including degree, betweenness, closeness, stress, bottleneck, eccentricity, radiality, clustering coefficient, edge percolated component (EPC), density of maximum neighborhood component (DMNC), and maximal clique centrality (MCC). Genes were ranked according to each algorithm, and the top 30 genes were selected for visualization.

The PPI networks and the top 30 hub genes ranked by degree centrality for the magenta and black modules are shown in Figure 3E–H. Overall, 341 hub genes were identified from the GEO (magenta, pink, tan, and yellow modules) (Table S3) and 264 hub genes were determined from the TCGA (black and green modules) (Table S4). Several hub genes, including S100A9, have been reported as important regulators of inflammatory signaling, myeloid differentiation, and tumor‐promoting microenvironments. Their central positions within the interaction networks suggest potential roles as key coordinators of immune and metabolic pathways involved in leukemogenesis. Detailed PPI network information for the remaining modules is presented in Figures [Link], [Link], [Link], [Link], [Link], [Link].

### 3.3 Integration With Metabolic Gene Sets

There were 948 metabolic genes from MSigDB, 341 unique genes from all modules of GEO data, and 264 unique genes from all modules of TCGA data. As shown in Figure 3I, intersection with MSigDB metabolic genes yielded 35 metabolic‐related hub genes entirely, representing candidates linking metabolic alterations with AML pathogenesis.

### 3.4 Internal Validation and Construction of Prognostic Risk Model

**FIGURE 4:**
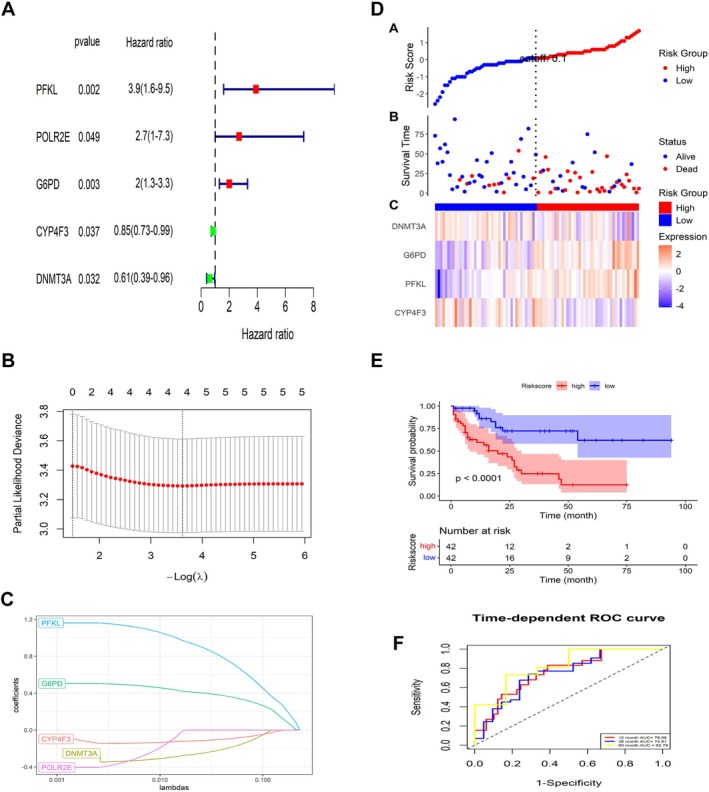
Development and validation of a metabolic‐related prognostic signature in AML. (A) Univariable Cox regression analysis in the TCGA‐LAML training cohort identified genes significantly associated with OS (*p* < 0.05), which were retained as candidates for model construction. (B) LASSO Cox regression was applied to reduce dimensionality and prevent overfitting; coefficient trajectories across log(λ) illustrate the shrinkage and selection process. (C) Ten‐fold cross‐validation was used to determine the optimal λ value based on the minimum partial likelihood deviance and the 1‐standard‐error criterion. (D) The relationship between the expression of four metabolic signatures and risk score, overall survival, and living status. (E) Kaplan–Meier survival analysis showed significantly poorer OS in the high‐risk group compared with the low‐risk group. (F) Time‐dependent ROC curves at 12, 36, and 60 months demonstrated the predictive performance and stability of the risk model. AML, acute myeloid leukemia; LASSO, least absolute shrinkage and selection operator; OS, overall survival; ROC, receiver operating characteristic; TCGA, The Cancer Genome Atlas.

To investigate the predictive performance of final hub genes, 80% of the total sample of TCGA‐LAML data was selected for the internal validation of the train set. Five MRPGs were initially identified by univariable Cox analysis (Figure 4A), and four genes: PFKL, G6PD, DNMT3A, and cytochrome P450 family 4 subfamily F member 3 (CYP4F3) were retained by LASSO (Figure 4B,C).

The distribution of risk scores and survival status is shown in Figure 4D. Kaplan–Meier analysis indicated significantly poorer survival in the high‐risk group (*p* < 0.0001; Figure 4E). Furthermore, the model demonstrated strong predictive performance (AUC: 0.76, 0.75, 0.83 at 1‐, 3‐, and 5‐year, Figure 4F). Notably, PFKL and G6PD were associated with poor prognosis, consistent with their roles in glycolysis and redox metabolism, while DNMT3A and CYP4F3 showed protective associations.

### 3.5 Construction of Nomogram and Evaluation of Clinical Data

**FIGURE 5:**
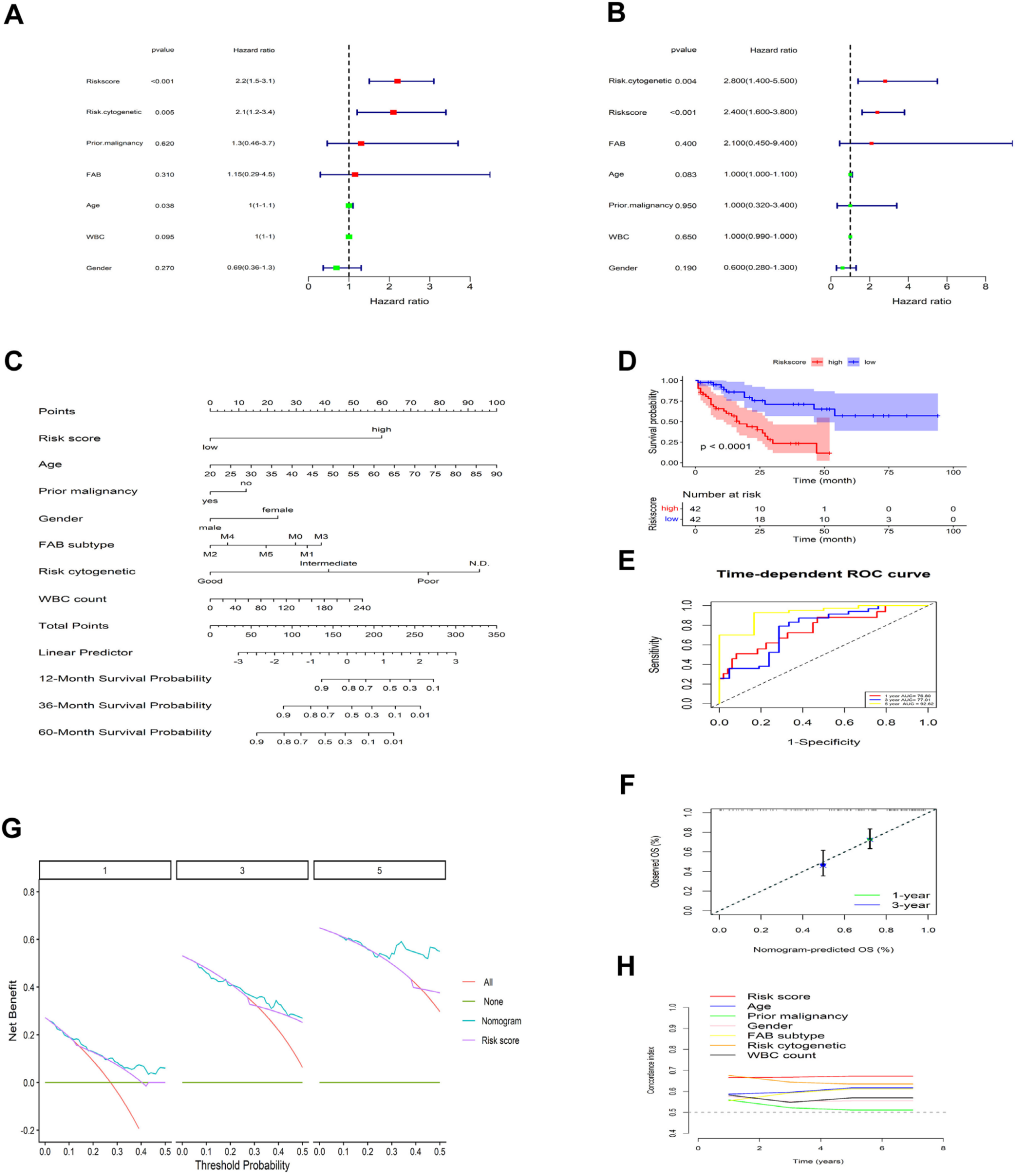
Clinical prognostic analysis and nomogram construction in the training set. (A) Univariable Cox regression analysis of OS evaluating risk score, risk cytogenetic, prior malignancy, FAB subtype, age, WBC count, and gender. (B) Multivariable Cox regression analysis including all variables, identifying the risk score and risk cytogenetic as independent prognostic factors. (C) Nomogram integrating the risk score and clinical variables (age, gender, FAB subtype, prior malignancy, risk cytogenetic, and WBC count) to estimate 1‐, 3‐, and 5‐year OS probabilities, providing a quantitative tool for individualized prognostication in AML. (D) Kaplan–Meier survival curves for nomogram‐derived risk groups. Patients stratified by the composite nomogram score exhibited significantly different OS outcomes (*p* < 0.0001), supporting the model's prognostic value. (E) ROC curves at 1, 3, and 5 years demonstrate the discriminative performance of the nomogram, indicating robust predictive accuracy across time points. (F) Calibration plots at 1 and 3 years comparing predicted and observed survival probabilities. The curves closely align with the 45° reference line, indicating good calibration. (G) DCA at 1, 3, and 5 years shows that the nomogram provides greater net clinical benefit across a wide range of threshold probabilities compared with the metabolic risk score alone and default strategies (treat‐all or treat‐none). (H) Time‐dependent C‐index curves comparing prognostic performance of individual variables. The metabolic gene–derived risk score consistently achieves the highest C‐index, indicating strong independent prognostic contribution. C‐index, concordance index; DCA, decision curve analysis; FAB, French‐American‐British classification; OS, overall survival; ROC, Receiver operating characteristic; WBC, white blood cell.

The prognostic value of the metabolic risk score was assessed against conventional clinical variables in AML using the training dataset. In univariable Cox analysis, the risk score and risk cytogenetic were significantly associated with OS, while age showed a borderline effect (Figure 5A). In multivariable analysis, both the risk score (HR≈2.4) and risk cytogenetic (HR≈2.8) remained independent predictors of poor survival (Figure 5B). A nomogram was constructed to integrate the calculated risk score with each clinical data (age, prior malignancy, gender, risk cytogenetic, French‐American‐British (FAB) subtype, and white blood cell count (WBC)) (Figure 5C). These variables demonstrated strong associations with OS in earlier analyses. Kaplan–Meier curves showed that patients in the high‐risk group had significantly poorer OS compared with those in the low‐risk group (Figure 5D).

ROC curve analysis further confirmed the strong predictive performance of the model, yielding AUC values of 76.80%, 77.01%, and 92.62% for 1‐, 3‐, and 5‐year survival, respectively (Figure 5E). Calibration plots for the 1‐year and 3‐year time points demonstrated good alignment between predicted and observed outcomes, closely following the ideal reference line (Figure 5F). Although slight deviations were noted at higher predicted probabilities, these are likely attributable to limited sample size and increased censoring at later follow‐up. Overall, the nomogram displayed satisfactory calibration for short‐ and mid‐term survival prediction.

**FIGURE 6:**
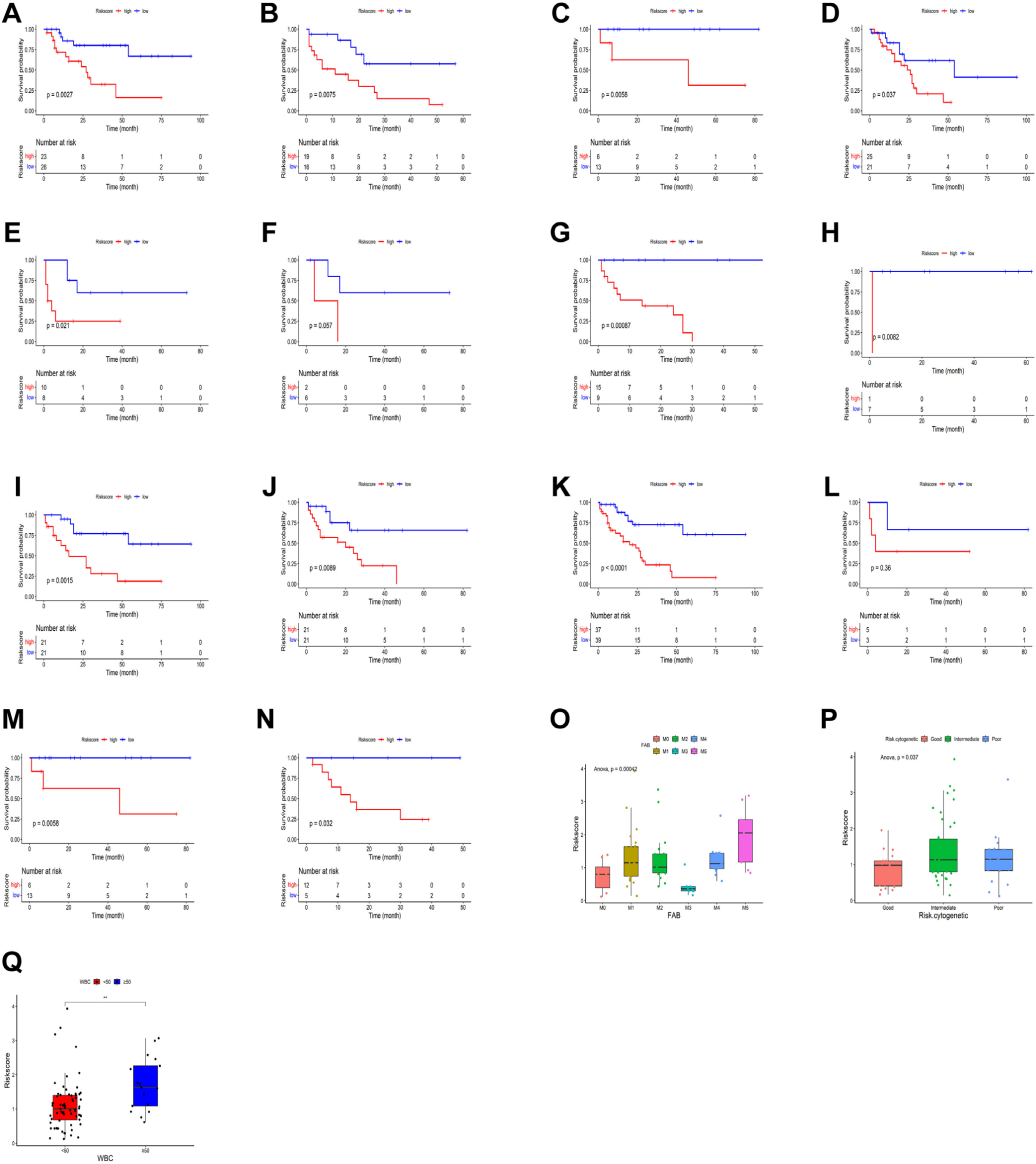
Association of clinical characteristics with overall survival and their distribution in the train set. (A–N) Kaplan–Meier analyses of OS according to clinical characteristics in the train set. Patients were stratified by age (< 60 [A] vs. ≥ 60 years [B]); risk cytogenetic (good [C], intermediate [D], poor [E]); FAB subtype (M0 [F], M1 [G], M3 [H]); gender (male [I], female [J]); prior malignancy (no [K], yes [L]); and WBC count (< 50 [M] vs. ≥ 50 [N]). Survival differences were assessed using the log‐rank test. (O–Q) Distribution of clinical variables and risk score across patient groups. (O) Boxplot of risk score across FAB subtypes. (P) Risk score distribution among risk cytogenetic categories. (Q) Comparison of WBC levels between groups. Statistical significance was assessed using nonparametric tests, with *p*‐values indicated. These analyses further illustrate the relationships between clinical features, risk stratification, and patient prognosis in AML. FAB, French–American–British classification; OS, overall survival; WBC, white blood cell.

Decision curve analysis (Figure 5G) indicated that the nomogram provided greater clinical benefit than individual predictors and the risk score alone across 1‐, 3‐, and 5‐year time horizons. Across a broad range of threshold probabilities, the nomogram consistently achieved higher net benefit compared with the default strategies of treating all or treating no patients. The greatest net benefit was observed at the 1‐year time point, suggesting an improved ability to identify high‐risk individuals who may require closer monitoring or intensified treatment. Similar trends were maintained at 3 and 5 years. As shown in Figure 5H, the C‐index of the variables in the training cohort indicates favorable discriminative ability and supports the reliability of the model. Collectively, these results demonstrate that the constructed nomogram is an effective and robust tool for predicting overall survival in AML patients. In addition, Kaplan–Meier analyses of each clinical data showed that older age, poor‐risk cytogenetics, certain FAB subtypes, and high WBC count were associated with worse survival (Figure 6A–N). Boxplot analysis further showed that higher risk scores were associated with adverse FAB subtypes, poor cytogenetic categories, and elevated WBC levels (Figure 6O–Q). Overall, these findings indicate that the metabolic‐related risk score provides additional prognostic value beyond traditional clinical markers in AML.

### 3.6 Validation of the Risk Model and Metabolic‐Related Prognostic Signatures

**FIGURE 7:**
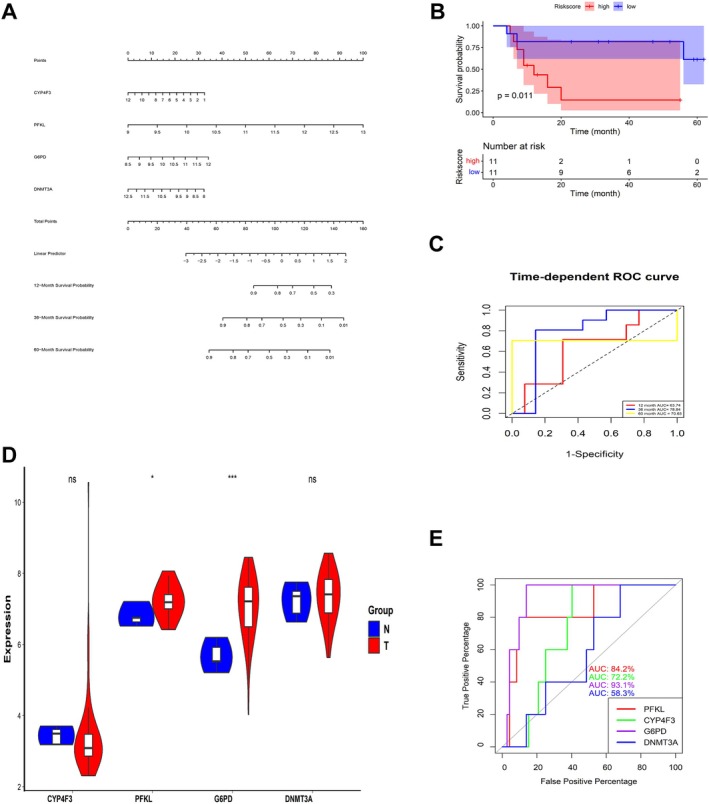
Nomogram construction, internal validation of the prognostic model, and external gene‐level evaluation in GSE68172. (A) Nomogram for predicting overall survival in patients based on the expression levels of four hub genes (CYP4F3, PFKL, G6PD, and DNMT3A). The model integrates gene expression‐derived risk contributions to estimate the probability of 1‐, 3‐, and 5‐year survival. Each variable corresponds to a point value, and the total score is used to calculate the predicted survival probability. (B) Kaplan–Meier analysis of OS in the test cohort. Patients stratified into high‐ and low‐risk groups based on the metabolic gene‐derived risk score showed significantly poorer OS in the high‐risk group, supporting model generalizability. (C) Time‐dependent ROC curves for OS prediction at 12, 36, and 60 months in the test cohort, indicating stable discriminative performance. (D) Boxplots of MRPGs expression in AML and normal samples (GSE68172). Expression of CYP4F3, PFKL, G6PD, and DNMT3A was compared between AML (T) and normal (N) groups. G6PD was significantly upregulated in AML (***p < 0.001), and PFKL showed a modest increase (*p < 0.05), whereas CYP4F3 and DNMT3A were not significantly different (ns). (E) ROC analysis of MRPGs in GSE68172. G6PD (AUC = 93.1) and PFKL (AUC = 84.2) showed stronger discriminatory performance than CYP4F3 and DNMT3A, supporting their potential diagnostic relevance in AML. AML, acute myeloid leukemia; AUC, area under the curve; CYP4F3, cytochrome P450 family 4 subfamily F member 3; DNMT3A, DNA methyltransferase 3 alpha; G6PD, glucose‐6‐phosphate dehydrogenase; MRPGs, metabolic‐related prognostic genes; ns, not significant; OS, overall survival; PFKL, phosphofructokinase liver type; ROC, receiver operating characteristic.

To provide a practical tool for clinical prognosis, a nomogram was developed incorporating the expression levels of the four hub genes (CYP4F3, PFKL, G6PD, and DNMT3A) to estimate 1‐, 3‐, and 5‐year survival probabilities (Figure 7A). The performance of the prognostic risk model was then assessed in the internal test set. Kaplan–Meier analysis demonstrated that patients in the high‐risk group had significantly shorter overall survival than those in the low‐risk group (*p* < 0.011; Figure 7B). Time‐dependent ROC analysis further supported the predictive value of the model, with AUCs of 63.74, 78.84, and 70.65 for 1‐, 3‐, and 5‐year overall survival, respectively (Figure 7C).

To further assess the relevance of the individual hub genes in an independent dataset, we analyzed GSE68172. Boxplot analysis showed that although CYP4F3 and DNMT3A were upregulated in AML samples relative to normal controls, these differences were not statistically significant. In contrast, G6PD expression was significantly elevated in AML (***p < 0.001), and PFKL showed a moderate but significant increase (*p < 0.05) (Figure 7D). ROC analysis of the individual genes in GSE68172 yielded AUCs of 84.2 for PFKL, 72.2 for CYP4F3, 93.1 for G6PD, and 58.3 for DNMT3A (Figure 7E). Collectively, these findings support the prognostic utility of the internally validated risk model and provide independent gene‐level evidence for the relevance of several metabolic biomarkers in AML.

### 3.7 Biological Characterization of Risk Groups

**FIGURE 8:**
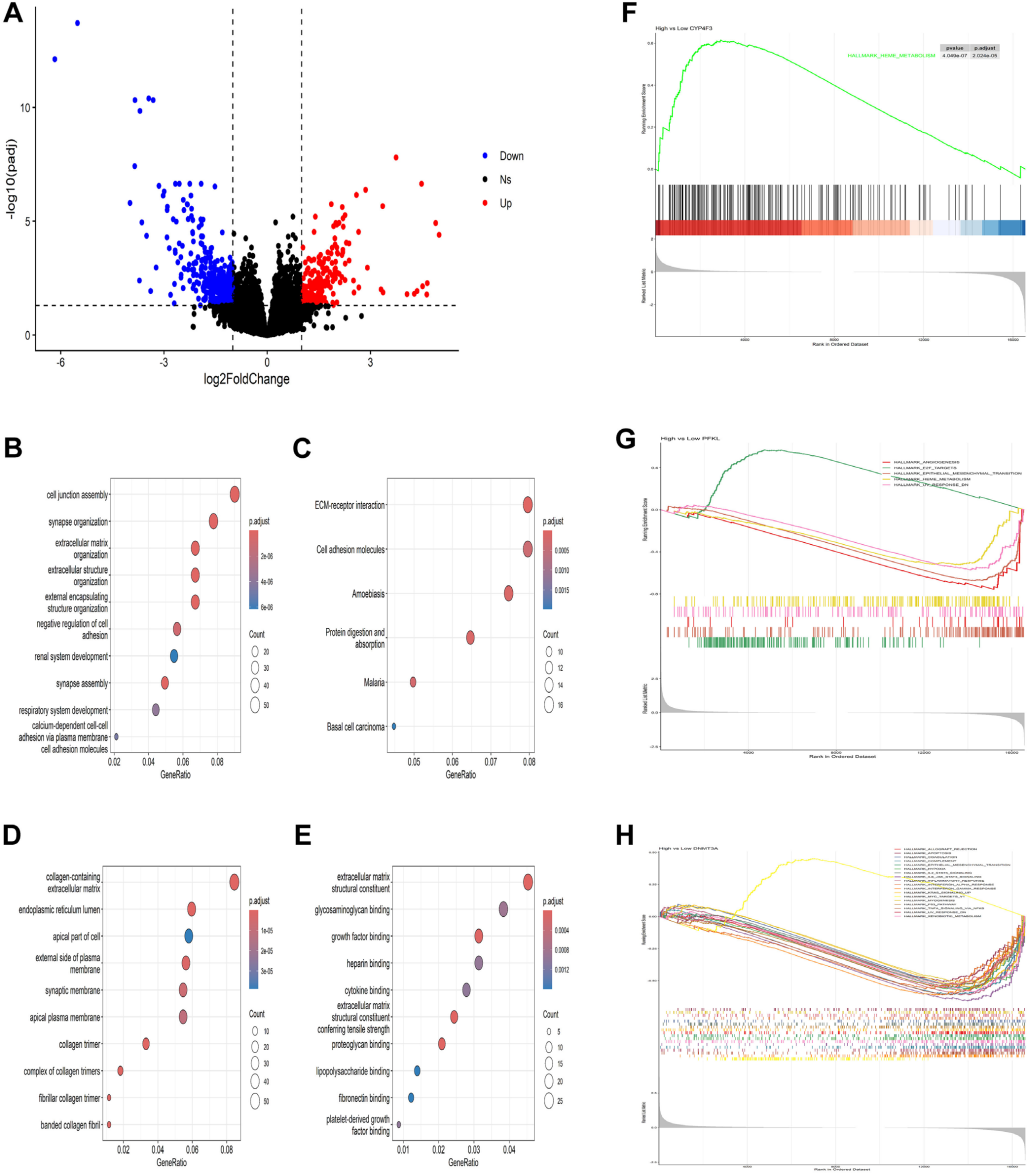
Functional enrichment and pathway analysis associated with the MRPGs. (A) Volcano plot of DEGs between high‐ and low‐risk groups in the TCGA‐LAML training cohort. Red and blue dots indicate significantly upregulated and downregulated genes, respectively (|log_2_FC| > 1, adjusted *p* < 0.05). (B) GO of BP enrichment analysis of DEGs using the clusterProfiler package. Enriched terms were primarily related to extracellular matrix organization, cell junction assembly, and structural organization, suggesting alterations in cell adhesion and the tumor microenvironment. (C) KEGG pathway enrichment analysis of DEGs. Significantly enriched pathways included focal adhesion, ECM–receptor interaction, PI3K–Akt signaling, and cytokine‐cytokine receptor interaction, indicating dysregulation of metabolic and signaling pathways in high‐risk AML. (D–F) GSEA of selected MRPGs. (D) CYP4F3 and (E) PFKL were mainly associated with metabolic and oxidative stress‐related pathways, whereas (F) DNMT3A was linked to epigenetic regulation and differentiation processes. GSEA results for G6PD did not meet visualization thresholds and are not shown. AML, acute myeloid leukemia; BP, biological process; DEGs, differentially expressed genes; GO, Gene Ontology; GSEA, gene set enrichment analysis; KEGG, Kyoto Encyclopedia of Genes and Genomes; MRPGs, metabolic‐related prognostic genes.

Differential expression analysis between high‐ and low‐risk groups in the TCGA cohort identified 641 DEGs (|log_2_FC| > 1, *p* < 0.05), which were visualized in a volcano plot (Figure 8A). KEGG analysis showed enrichment in pathways related to extracellular matrix remodeling and cell interaction, including ECM‐receptor interaction and cell adhesion molecules, suggesting increased invasiveness and microenvironment remodeling in the high‐risk group (Figure 8B). Metabolic pathways such as protein digestion and absorption were also enriched, consistent with the metabolism‐based gene selection strategy. In addition, cancer‐ and infection‐related pathways were identified, reflecting oncogenic, immune, and inflammatory activities. GO analysis further indicated enrichment in extracellular matrix organization, cell adhesion, immune response, and membrane‐associated components (Figure 8C–E). Together, these findings suggest that the high‐risk group is characterized by tumor microenvironment remodeling, immune dysregulation, and metabolic alterations.

### 3.8 Functional Enrichment Analysis of MRPGs

GSEA was performed using the TCGA‐LAML dataset to investigate pathways associated with MRPGs expression. G6PD showed no significant enrichment and was therefore excluded from further analysis. High CYP4F3 expression was associated with heme metabolism (Figure 8F). High and low PFKL expression were enriched in E2F targets and angiogenesis, respectively (Figure 8G). In addition, high DNMT3A expression was associated with MYC target signaling related to cell growth and metabolism, whereas low DNMT3A expression was associated with hypoxia, immune signaling, and inflammatory response pathways (Figure 8H). These results indicate that MRPGs are linked to immune modulation, metabolic activity, and oncogenic signaling in AML.

## 4 Discussion

Recent advances in AML biology and mutational profiling have enabled the development of targeted therapies, yet the mechanisms driving leukemogenesis remain incompletely understood [18]. Metabolic deregulation has emerged as a key driver of AML progression, therapeutic resistance, immune dysregulation, and the maintenance of leukemic stem cells [18, 19, 20, 21, 22, 23, 24]. In this study, we integrated co‐expression network analysis from GEO and TCGA datasets with curated metabolic gene sets to identify a metabolic‐related prognostic signature. This approach yielded a four‐gene model (PFKL, G6PD, CYP4F3, and DNMT3A) that consistently stratified patients into distinct risk groups across multiple cohorts.

Each gene in the signature reflects a complementary aspect of AML metabolic or regulatory biology. PFKL, a key glycolytic enzyme, aligns with the concept that enhanced glycolytic flux supports leukemic proliferation and metabolic adaptation to cellular stress [25]. Its enrichment in angiogenesis and proliferation‐related pathways and its relevance in several cancers [26, 27, 28, 29, 30] reinforce its biological significance. G6PD, the rate‐limiting enzyme of the pentose phosphate pathway, supports NADPH production and redox balance, both of which are essential for AML survival under oxidative stress [31, 32]. Redox homeostasis and the detoxification of reactive oxygen species are essential for leukemic cell survival, as supported by the elevated G6PD expression observed in the external validation cohort. Moreover, the documented immunogenic potential of G6PD in malignancies highlights its possible role in shaping the AML immune microenvironment [33]. Conversely, CYP4F3 and DNMT3A showed protective associations in the expression‐based model. The CYP4F3 gene encodes two isoforms, CYP4F3A and CYP4F3B, that perform distinct functions. CYP4F3A acts as an inflammatory modulator by inactivating leukotriene B4 (LTB4), whereas CYP4F3B converts arachidonic acid into 20‐hydroxyeicosatetraenoic acid (20‐HETE), an important mediator of neurogenic and vascular inflammation [34]. Mutations in CYP4F3 have been attributed to lung cancer development [35]. In addition, its upregulation has been observed in the AML cell line HL‐60 following treatment with all‐trans‐retinoic acid (ATRA) [36]. As shown in the risk heatmap, CYP4F3 expression was higher in the low‐risk group and lower in the high‐risk group, suggesting that increased CYP4F3 expression is associated with a better prognosis. Heme metabolism was considerably enriched in samples with high CYP4F3 expression (*p* < 0.001), according to gene set enrichment analysis. This finding implies that CYP4F3 may be involved in heme‐related metabolic regulation associated with favorable clinical outcomes. In accordance with these data, Wen J. and colleagues demonstrated that reduced activity of the CYP4F3 metabolic axis corresponds to worse outcomes [37]. Our model represents gene expression rather than mutational status, which may account for the varied behavior even though DNMT3A mutations are frequently associated with a bad prognosis [38, 39]. This is because DNMT3A mutations change DNA methylation activity regardless of expression levels. Importantly, pathway enrichment analysis of DEGs consistently implicated immune processes and extracellular matrix remodeling, indicating that metabolic risk may capture both tumor‐intrinsic and microenvironmental alterations. This is in accordance with Tien et al.'s research on AML [40]. In their study, bulk bone marrow RNA sequencing confirmed that the immune‐related pathways and metabolic hallmarks, including oxidative phosphorylation, were associated with patient prognosis. Clinically, the metabolic risk score remained an independent predictor of overall survival after adjusting for established prognostic factors. This is consistent with prior reports demonstrating that expression‐based or metabolic‐related signatures provide added prognostic value in AML [20, 41, 42]. The nomogram integrating the risk score with clinical variables demonstrated predictive performance, indicating a potential utility for individualized outcome estimation [43, 44]. Furthermore, patients with unfavorable FAB subtypes, high WBC counts, and unfavorable cytogenetic characteristics were more likely to have high metabolic risk scores. This finding demonstrated that metabolic dysregulation may be linked to aggressive disease biology and chromosomal instability [45]. However, the integrated nomogram was constructed in the training cohort and evaluated in the internal test cohort, while the external GSE68172 dataset was used for validation of individual hub genes rather than the complete prognostic model. These results imply that the signature captures a metabolically reprogrammed, high‐risk phenotype that interacts mechanistically with traditional prognostic markers. Furthermore, they may contribute to future improvements in AML risk stratification frameworks, such as the current European LeukemiaNet (ELN)‐based classification systems [46].

There are several limitations to this study. This retrospective research relied on publicly accessible datasets with variable treatment regimens. Moreover, the signature is expression‐based and did not incorporate CRISPR dependency analyses, drug‐sensitivity data, and mutational profiles (including DNMT3A). Additionally, experimental validation will be required to determine the relationships between the identified genes and leukemogenesis, especially in the biology of AML stem cells and metabolic adaptation mechanisms. To ascertain whether focusing on glycolysis, the activity of the pentose phosphate pathway, or associated metabolic pathways could enhance outcomes in specific AML subgroups, future studies integrating multi‐omics data, prospective clinical validation, and mechanistic research will be necessary.

## 5 Conclusions

In this study, a four‐gene metabolic‐related prognostic signature (PFKL, G6PD, CYP4F3, and DNMT3A) was identified and validated. This signature robustly stratifies AML patients into distinct risk groups and provides prognostic value beyond conventional clinical and cytogenetic factors. This signature reflects key aspects of metabolic reprogramming, immune regulation, and tumor microenvironment remodeling in AML. Integration of the signature into a prognostic nomogram enabled accurate, individualized survival predictions. Overall, our findings highlight the critical role of metabolic pathways in AML progression and prognosis and support the potential clinical utility of metabolism‐informed risk assessment. Nevertheless, prospective clinical validation and further mechanistic studies are needed to clarify the biological functions of these genes and to determine whether targeting metabolic vulnerabilities can improve therapeutic outcomes in AML.

## Author Contributions

**Reza Valadan:** writing – review and editing, supervision. **Hossein Asgarian‐Omran:** conceptualization, funding acquisition, project administration, validation, writing – review and editing, methodology. **Fatemeh Akhoundi:** methodology, writing – review and editing, investigation. **Samaneh Ramezani:** conceptualization, funding acquisition, project administration, validation, writing – review and editing, methodology.

## Funding

This research was supported by the Mazandaran University of Medical Sciences under Grant (No. 20046).

## Ethics Statement

The authors have nothing to report.

## Conflicts of Interest

The authors declare no conflicts of interest.

## Supporting information


**Figure S1:** PCA and clustering analyses of GEO and TCGA AML datasets. (A, B) PCA of GEO samples before and after batch‐effect correction. (C) Hierarchical clustering of GEO samples for outlier detection. (D) Hierarchical clustering of uniformly processed TCGA‐LAML samples from Recount2, used without batch correction. PCA, principal component analysis; GEO, Gene Expression Omnibus; TCGA, The Cancer Genome Atlas.

**Figure S2:** Sample clustering and module eigengene heatmaps in GEO and TCGA AML datasets. (A) Sample dendrogram and trait heatmap (NT: normal vs. tumor) for GEO data. (B) TOM heatmap showing gene network connectivity in GEO. (C) Sample dendrogram and trait heatmap for TCGA‐LAML data. (D) TOM heatmap illustrating gene co‐expression structure in TCGA. (E, F) Heatmaps of module eigengenes showing relationships among identified modules in GEO and TCGA datasets. GEO, Gene Expression Omnibus; TCGA, The Cancer Genome Atlas; TOM, Topological overlap matrix.

**Figure S3:** Functional enrichment of hub modules in GEO data. (A–D) CC and MF enrichment for magenta (A, B) and green (C, D) modules. (E–H) BP, CC, MF, and KEGG enrichment for the pink module. Dot size corresponds to gene count, and color indicates the *p*‐value. GEO, Gene Expression Omnibus; CC, cellular component; MF, molecular function; BP, biological process; KEGG, Kyoto Encyclopedia of Genes and Genomes.

**Figure S4:** Functional enrichment of hub modules in GEO and TCGA datasets. (A–D) BP, CC, MF, and KEGG enrichment for the GEO tan module. (E–G) BP, CC, and MF enrichment for the GEO yellow module; KEGG pathways were not significantly enriched and therefore are not shown. (H–K) BP, CC, MF, and KEGG enrichment for the TCGA black module. Dot size represents gene count and color indicates adjusted *p*‐value. GEO, Gene Expression Omnibus; TCGA, The Cancer Genome Atlas; CC, cellular component; MF, molecular function; BP, biological process; KEGG, Kyoto Encyclopedia of Genes and Genomes.

**Figure S5:** PPI network and CytoHubba hub gene analysis for the magenta module. (A–J) Top 30 genes ranked by 11 CytoHubba algorithms for the magenta module (network and degree are shown in Figure 3E,G). Node color reflects degree centrality, with darker colors indicating higher connectivity. PPI, Protein–protein interaction.

**Figure S6:** PPI network and CytoHubba hub gene analysis for the pink module. (A–L) Top 30 genes ranked by 11 CytoHubba algorithms for the pink module. Node color reflects degree centrality, with darker colors indicating higher connectivity. PPI, Protein–protein interaction.

**Figure S7:** PPI network and CytoHubba hub gene analysis for the tan module. (A–L) Top 30 genes ranked by 11 CytoHubba algorithms for the tan module. Node color reflects degree centrality, with darker colors indicating higher connectivity. PPI, Protein–protein interaction.

**Figure S8:** PPI network and CytoHubba hub gene analysis for the yellow module. (A–L) Top 30 genes ranked by 11 CytoHubba algorithms for the yellow module. Node color reflects degree centrality, with darker colors indicating higher connectivity. PPI, Protein–protein interaction.

**Figure S9:** PPI network and CytoHubba hub gene analysis for the green module. (A–L) Top 30 genes ranked by 11 CytoHubba algorithms for the green module. Node color reflects degree centrality, with darker colors indicating higher connectivity. PPI, Protein–protein interaction.

**Figure S10:** PPI network and CytoHubba hub gene analysis for the black module. (A–J) Top 30 genes ranked by 11 CytoHubba algorithms for the black module (network and degree are shown in Figure 3F,H). Node color reflects degree centrality, with darker colors indicating higher connectivity. PPI, Protein–protein interaction.

**Table S1:** Number of genes in the final selected modules identified from the GEO and TCGA datasets.

**Table S2:** Gene symbols included in all co‐expression modules identified from the GEO and TCGA datasets using WGCNA.

**Table S3:** Top 30 genes in the STRING protein–protein interaction networks ranked by 11 CytoHubba methods for magenta, pink, tan, and yellow modules in the GEO dataset.

**Table S4:** Top 30 genes in the STRING protein–protein interaction networks ranked by 11 CytoHubba methods for green and black modules in the TCGA dataset.

## Data Availability

The data that support the findings of this study are available from the corresponding author upon reasonable request.
